# PREVALENCE OF ELDER ABUSE AND NEGLECT OF PERSONS WITH DEMENTIA: A SYSTEMATIC REVIEW AND META-ANALYSIS

**DOI:** 10.1093/geroni/igad104.2129

**Published:** 2023-12-21

**Authors:** Fei Sun, Xuehan Zhang, Yan Shen, Yali Feng, Teri Kennedy

**Affiliations:** Michigan State University, East Lansing, Michigan, United States; Michigan State University, East Lansing, Michigan, United States; Hubei Normal University, Huangshi, Hubei, China (People’s Republic); University of Illinois, Urbana, Illinois, United States; University of Kansas Medical Center, Kansas City, Kansas, United States

## Abstract

People with dementia (PWD) are at high risk for abuse and neglect. Little is known about the synthesized prevalence rate of abuse among this population though. This study reviewed articles on abuse and neglect among PWD to learn about the pooled prevalence estimates of elder abuse among this population with a meta-analytic approach and examined the heterogeneity associated with the prevalence estimates through meta-regression and subgroup analyses. A total of 27 studies were selected from 479 relevant studies after careful identification using eight academic databases (e.g., ProQuest, CINAHL Plus). All 27 studies received acceptable scores on study quality and demonstrated no significant publication biases. All the studies included a total sample of 11,246 care recipient and caregiver dyads. In addition to overall abuse, three subtypes of elder abuse including physical abuse, emotional abuse, and neglect were examined. Results showed that the pooled prevalence of overall elder abuse was 41% (95%CI [0.31,0.49], p < 0.001). The pooled prevalence of abuse subtypes were 43%(95% CI [0.35, 0.52], p < .001) for emotional abuse, followed by neglect at 16%, and physical abuse at10% (95% CI [0.07, 0.13], p < 0.01). Overall, studies with caregiver average age below 60. reported higher abuse rates than those with caregivers’ age over 60. These results call for more policy and practice efforts to assist PWD who are victims of abuse and neglect, and more so, to prevent caregivers from exerting abuse and neglect behaviors toward PWD via education, skills training and support.

